# ST-YOLOA: a Swin-transformer-based YOLO model with an attention mechanism for SAR ship detection under complex background

**DOI:** 10.3389/fnbot.2023.1170163

**Published:** 2023-06-02

**Authors:** Kai Zhao, Ruitao Lu, Siyu Wang, Xiaogang Yang, Qingge Li, Jiwei Fan

**Affiliations:** Department of Automation, Rocket Force University of Engineering, Xi'an, China

**Keywords:** synthetic aperture radar (SAR) image, ship detection, Swin Transformer, YOLO, attention mechanism

## Abstract

A synthetic aperture radar (SAR) image is crucial for ship detection in computer vision. Due to the background clutter, pose variations, and scale changes, it is a challenge to construct a SAR ship detection model with low false-alarm rates and high accuracy. Therefore, this paper proposes a novel SAR ship detection model called ST-YOLOA. First, the Swin Transformer network architecture and coordinate attention (CA) model are embedded in the STCNet backbone network to enhance the feature extraction performance and capture global information. Second, we used the PANet path aggregation network with a residual structure to construct the feature pyramid to increase global feature extraction capability. Next, to cope with the local interference and semantic information loss problems, a novel up/down-sampling method is proposed. Finally, the decoupled detection head is used to achieve the predicted output of the target position and the boundary box to improve convergence speed and detection accuracy. To demonstrate the efficiency of the proposed method, we have constructed three SAR ship detection datasets: a norm test set (NTS), a complex test set (CTS), and a merged test set (MTS). The experimental results show that our ST-YOLOA achieved an accuracy of 97.37%, 75.69%, and 88.50% on the three datasets, respectively, superior to the effects of other state-of-the-art methods. Our ST-YOLOA performs favorably in complex scenarios, and the accuracy is 4.83% higher than YOLOX on the CTS. Moreover, ST-YOLOA achieves real-time detection with a speed of 21.4 FPS.

## 1. Introduction

SAR imaging has a high resolution, a long detection range, and a strong anti-interference ability. It has a wide range of applications and development possibilities and is employed extensively in both civil and military fields, including surveying, mapping, catastrophe monitoring, marine observation, and military reconnaissance (Cumming and Wong, 2005; Moreira et al., 2013). Research on ship detection technology, such as precise terminal guidance, operational effectiveness assessment, and identification of ship targets in strategic port areas, is crucial for both military and civilian applications. Although significant progress has been made in the past few decades, ship detection in SAR images remains a challenge due to shape deformation, pose variation, and background clutter.

Traditional ship detection methods employ machine learning models to distinguish the ship target from the background in SAR images. These methods usually include two primary processes: object detection and target identification (Lu et al., 2020a,b). The most popular models employed in the classic detection approach are the constant false-alarm rate (CFAR) (Robey et al., 1992) and its variant algorithms. These methods detect ships by creating a statistical distribution model of the background clutter. By employing a decomposition strategy, Gao et al. (2018) suggested a CFAR algorithm based on a generalized gamma distribution to enhance the signal-to-noise ratio of SAR images. Wang et al. (2017) pro-posed a constant false-alarm detector in the intensity space domain, which uses data correlation to detect targets and wake pixels. To best suit the information provided by the ship distribution map (Schwegmann et al., 2015) proposed a method for transforming a scalar threshold into a threshold manifold using the simulated annealing (SA) process. However, the traditional SAR ship detection model relies on artificial design feature selection, which leads to poor detection robustness and generalization ability. In addition, this kind of algorithm requires a high contrast between the target image and the background image and is not suitable for detecting ship targets in complex environments.

With the development of deep learning theory, convolutional neural networks have made significant advancements in the field of target recognition and display advanced performance in target detection. The deep learning detection algorithms can be roughly classified into two categories: one-stage methods and two-stage methods (Girshick et al., 2014). Two-stage object detection methods perform region generation to obtain pre-selected boxes and then use sample classification and regression with border positioning through the convolutional neural network. Representative methods include R-CNN (Girshick et al., 2014), Fast R-CNN (Girshick, 2015), and Faster R-CNN (Ren et al., 2015). These methods have high detection accuracy but low detection efficiency. One-stage object detection methods use the backbone feature extraction network to directly locate and classify the target. Typical detection methods are YOLO (Redmon et al., 2016), SSD (Liu et al., 2016), and CenterNet (Duan et al., 2019). Although these methods are fast, they are prone to false detection and missing detection compared with the two-stage detection methods.

Based on the current deep-learning-based target detection, in recent years, numerous researchers have developed advanced algorithms for SAR ship detection. To address the challenges of ship detection in maritime environments, Gao et al. (2022) proposed an enhanced YOLOv5 SAR ship detection method based on target augmentation. By constructing the feature enhancement Swin converter (FESwin) module and the adjacent feature fusion (AFF) module (Li et al., 2022), proposed a detection model suitable for the strong scattering, multi-scale, and complicated background of ship objects in SAR images. To provide a visual converter system based on context-federated representation learning appropriate for SAR ship detection (Xia et al., 2022), creatively combined CNNs with transformers. Although the aforementioned methods address the issues of multi-scale targets, huge noise clutter, and complicated backgrounds, the detection accuracy and the calculation speed still restrict application in the real world. Small islands and nearby sea structures are the reasons for false detection in com-plex backgrounds. In addition, the dense distribution of ships in the dock and the sea causes multiple targets to overlap, leading to the low accuracy of models in detecting targets.

Based on the aforementioned analysis, in this paper, we have proposed a novel ship detection model called ST-YOLOA, which is more suitable for the actual complex environment in SAR images. We chose Swin Transformer (Liu et al., 2021) and YOLOX (Ge et al., 2021) as our basic models. The main contributions of this paper are as follows: (1) Together data on ship target features, we have proposed the STCNet backbone network. This network effectively solves the problem of insufficient feature extraction caused by strong scattering in SAR images. It enhances the processing ability of feature information by obtaining more significant feature information in different environments. It also has excellent global information modeling capabilities of Swin Transformer. (2) We have built a novel feature pyramid network based on an enhanced PANet for profoundly fusing high-level and low-level features. This network solves the issues of local information interference and attention diversion by using semantic and localization information. To improve the detection accuracy, we have adopted binary trilinear interpolation up-sampling for maintaining the original data of the feature map. (3) To effectively reduce the impact of noise in the feature map on the detection accuracy, classification and regression are handled separately. We have used EIOU as the localization loss function to cope with the sample imbalance and enhance the model generalization ability.

The rest of the paper is organized as follows. In Section 2, we review the prior work related to the proposed ST-YOLOA. In Section 3, we provide details of the main components and methodology of the proposed ST-YOLOA. Section 4 introduces the experimental settings, results, and analysis. The conclusions of the paper are drawn in Section 6.

## 2. Related work

In this section, we briefly review the relevant literature regarding YOLO, Transformer, and the attention mechanism.

### 2.1. YOLO

The YOLO series is a typical network for one-stage detection. It handles object detection as a regression issue, where the bounding box coordinates and class probability are derived from picture pixels. YOLOX, as a typical representative of the YOLO series, has significant advantages in terms of speed and accuracy. Its essential modules include the Focus, the CSP bottleneck, SPP, PANet (Liu et al., 2018), and the decoupled detection head (Tian et al., 2019). The backbone of YOLOX is the CSP Darknet-53 (Bochkovskiy et al., 2020), which consists of several residual modules stacked one on top of the other. YOLOX uses PANet as the neck of the model, and the input is the three feature output layers output by the backbone network. It obtains features with richer semantic and localization information by feature fusion and sends them to the head for detection. At the head, YOLOX replaces the coupled detection head with the decoupled detection head using different branches for the classification and regression tasks, which significantly increases the convergence speed of the network. YOLOX eliminates the constraints of the original anchor (Zhang et al., 2020) of the YOLO series. The anchor-free mechanism substantially reduces the number of design parameters, maintaining effectiveness while significantly reducing time costs.

When the YOLO architecture is used for ship detection, it mainly has the following two disadvantages: (1) It has poor recognition where small target objects are concerned, and the positioning is inaccurate. (2) It lacks the ability to obtain global information on the image that can benefit the network in terms of accuracy and efficiency.

### 2.2. Transformer

Transformer (Vaswani et al., 2017) was initially applied in the field of natural language processing (NLP) and has proved to have many advantages. Transformer is not only powerful in modeling global contexts, but also excellent in establishing long-distance dependencies. With its rapid development in the field of NLP, Transformer has attracted widespread attention in the field of the computer vision field. Swin Transformer (ST) is considered the first successful attempt to bring it into computer vision. It enables the Transformer model to process images at different scales flexibly by applying a hierarchical structure similar to that of CNN. ST performs local self-attention calculations in the area of non-overlapping windows. It lowers the computational complexity of the number from a squared relationship to a linear relationship. Then it uses shifted window multi-head self-attention (SW-MSA) to achieve information interaction between non-overlapping windows.

As a general visual network, ST exhibits state-of-the-art performance in semantic segmentation, object detection, and image classification. However, ST has two clear drawbacks: (1) ST has a limited ability to encode contextual information and needs further improvement. (2) Because ST has more parameters than CNN, its training usually relies on a large number of training data.

### 2.3. Attention mechanism

Usually, attention mechanisms in the vision domain (Guo M. et al., 2022) include two types: spatial and channel. They extract better target features by assigning different weights to the feature points on the image. The spatial attention mechanism adds weights to the feature points containing object features in a single-channel feature map. On the other hand, the channel attention mechanism assigns more importance to feature channels containing component semantic information. Hu et al. (2018) proposed SENet, which analyzes the correlation between different feature channels and generates channel descriptions by fusing features across spatial dimensions, thus achieving selective emphasis on feature information and suppressing irrelevant feature information; Woo et al. integrated the feature channels and feature space between correlation proposed CBAM (Wang et al., 2018), which can focus on more profound feature semantic information; Wang et al. proposed CANet (Hou et al., 2021), which considers inter-channel relationships as well as location information over long distances, based on the spatial selectivity of the channel attention mechanism. In this paper, the different characteristics of SE, CBAM, and CA, are introduced into other model modules to improve the performance model further.

## 3. Methods

**Figure 1:**
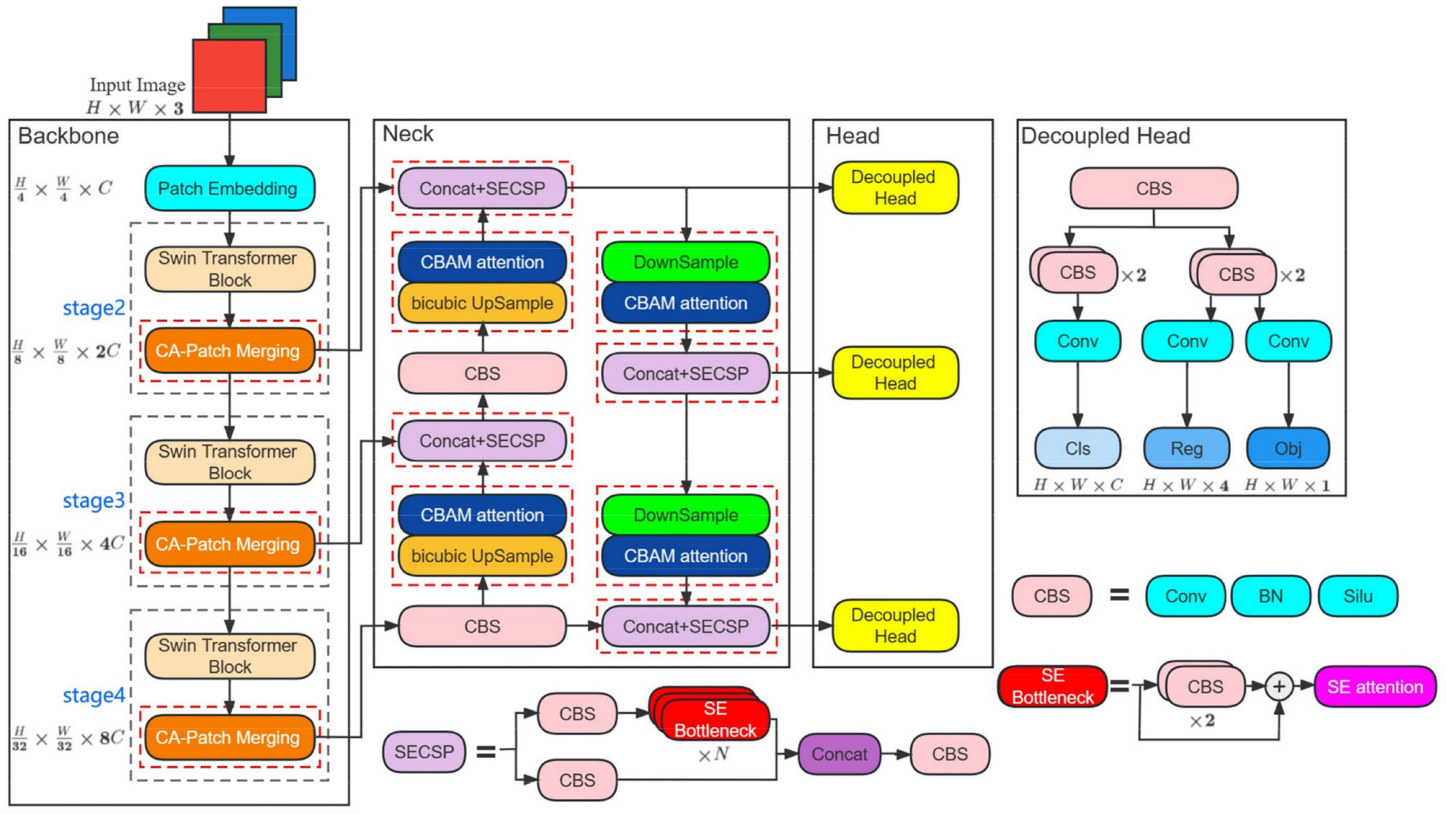
ST-YOLOA network structure. The red dashed boxes are the attention module addition locations. Conv, BN, and Silu denote the convolution, batch normalization, and SILU activation functions, respectively. Concat indicates the fully connected operation. Cls, reg, and obj represent the classification, localization, and confidence scores. *H, W*, and *C* denote the feature map's width, height, and number of channels.

In this section, we first give a general overview of the ST-YOLOA target detection model and then discuss in detail the design ideas and network architecture of the ST-YOLOA model in three parts: feature extraction (Backbone), feature fusion (Neck), and target detection (Head), respectively. Figure 1 shows the ST-YOLOA network structure.

### 3.1. Overview

#### 3.1.1. Backbone

In ST-YOLOA, we propose a backbone network called STCNet. It integrates the advantages of the Swin Transformer and the CA attention mechanism. Compared with the traditional CNN-based backbone feature extraction network, which only utilizes the information provided by regions in target localization, STCNet has good performance with dynamic attention and global modeling capability considering remote dependencies. The STCNet network adopts a layered architecture consisting of the Patch Embedding layer, the Swin Transformer Block, and the CA- Patch Merging layer composed of three parts.

#### 3.1.2. Neck

In the neck of ST-YOLOA, we still use PANet to construct feature pyramids (Lin et al., 2017a) for feature depth fusion. In addition, we also introduce SE and CBMA attention mechanisms in the neck to enhance the focus on the target information and further improve the model performance.

#### 3.1.3. Loss

The purpose of the loss function is mainly to make the model localization more accurate and recognition accuracy higher. Therefore, more advanced EIOU Loss is used in ST-YOLOA to accelerate the convergence and improve the model performance.

### 3.2. Backbone

#### 3.2.1. Patch embedding layer

**Figure 2:**
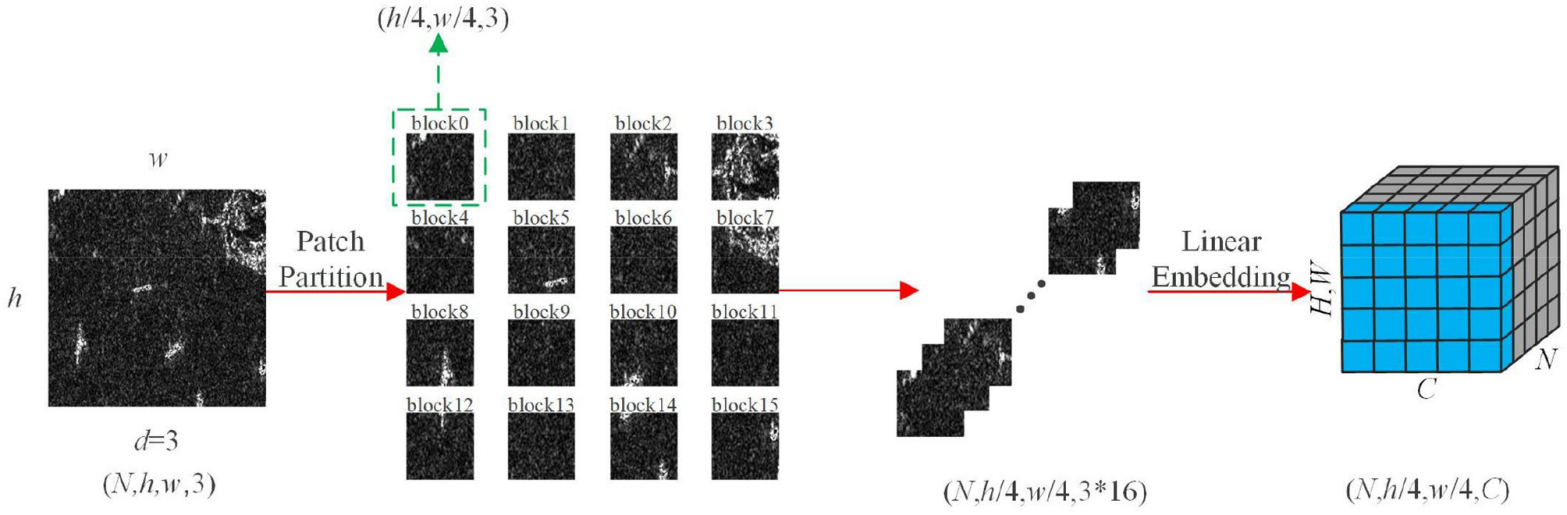
Patch Embedding structure. w and h are the length and width of the input feature map, d is the number of channel dimensions, and N is the batch size.

The patch embedding module first chunks the image at the front end of the feature extraction network, dividing the image into 4 × 4 non-overlapping blocks so that the feature dimension of each block is 4 × 4 × 3. Then, the original 2D image is converted into a series of 1D embedding vectors by projecting the feature dimensions to arbitrary dimensions through linear transformation, and the transformed embedding vectors are input to three-stage feature extraction layers to generate a hierarchical feature representation. The structure is shown in Figure 2.

#### 3.2.2. Swin transformer block

The Swin Transformer Block uses moving windows to calculate the attention between pixels, which helps to connect the front layer windows and reduce the complexity of the original attention calculation while overcoming the drawback of a lack of global effects, significantly enhancing the modeling effect.

**Figure 3:**
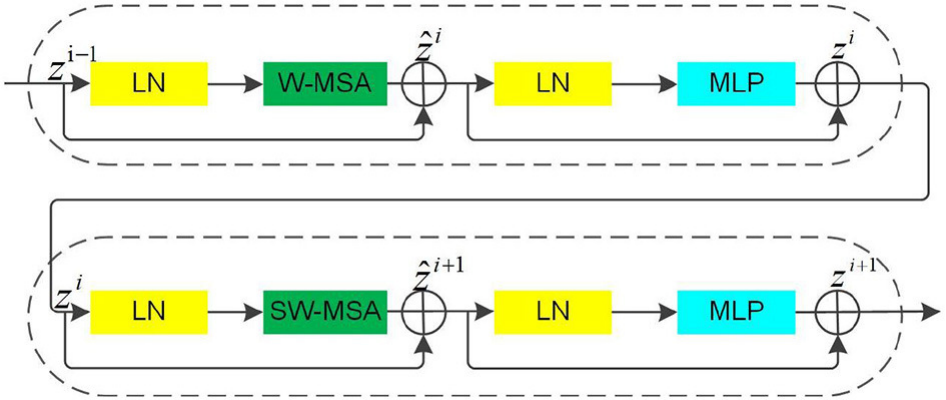
The Swin transformer blocks.

In Figure 3, it can be seen that the multiheaded self-attention (MSA) mechanism in the Swin Transformer Blocks is constructed based on the shift window. There are two consecutive Swin Transformer Blocks. Each Swin Transformer Block consists of a LayerNorm (LN) layer, an MSA module, a residual connection, and a multilayer perceptron (MLP) that contains two fully connected layers using the GELU non-linear activation function (Wang et al., 2021). The two consecutive Swin Transformer Blocks adopt the window multi-head self-attention (W-MSA) module and the shifted window multi-head self-attention (SW-MSA) module, respectively, which enables different windows to exchange information while reducing computational effort. Based on this window division mechanism, the continuous Swin Transformer Blocks are calculated as follows:


(1) $$\begin{array}{lll} {ẑ}^{i} & = & W-MSA\left(LN\left(z^{i-1}\right)\right)+z^{i-1} \end{array}$$

(2) $$\begin{array}{lll} z^{i} & = & MLP\left(LN\left({\hat{\text{z}}}^{i}\right)\right)+{ẑ}^{i} \end{array}$$

(3) $$\begin{array}{lll} {ẑ}^{i+1} & = & SW-MSA\left(LN\left(z^{i}\right)\right)+z^{i} \end{array}$$

(4) $$\begin{array}{lll} z^{i+1} & = & MLP\left(LN\left({ẑ}^{i+1}\right)\right)+{ẑ}^{i+1} \end{array}$$

Where ẑ^*i*^ denotes the output of the (S)W-MSA module and *z*^*i*^ denotes the output of the MLP module of the *i*^th^ Block.

#### 3.2.3. CA-patch merging

**Figure 4:**
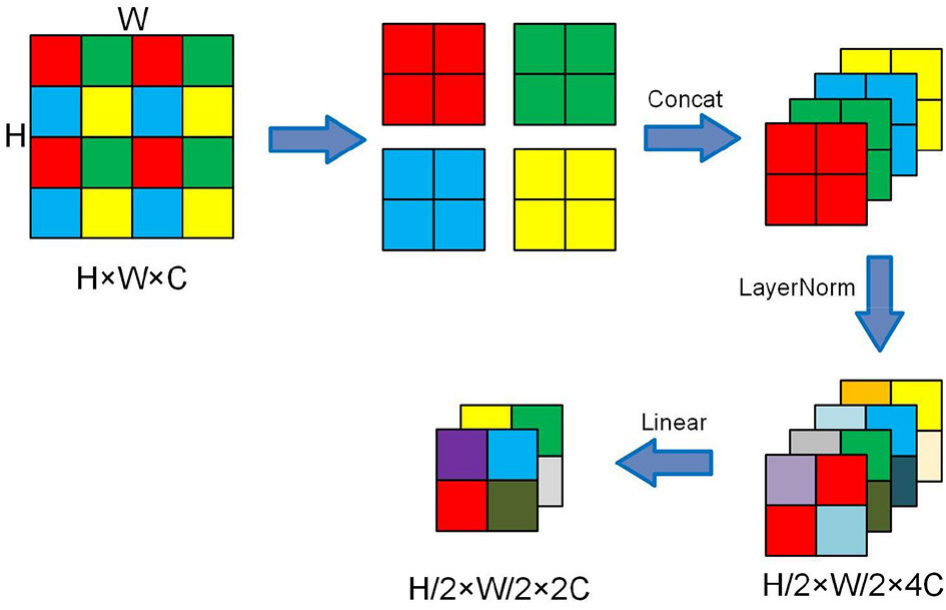
Schematic diagram of the Patch Merging layer.

The Patch Merging layer is used to perform a down-sample operation before the feature output of the backbone network to reduce the feature map resolution and adjust the number of channels, thus forming a layered design and also saving some computational effort. Figure 4 presents the working process.

**Figure 5:**
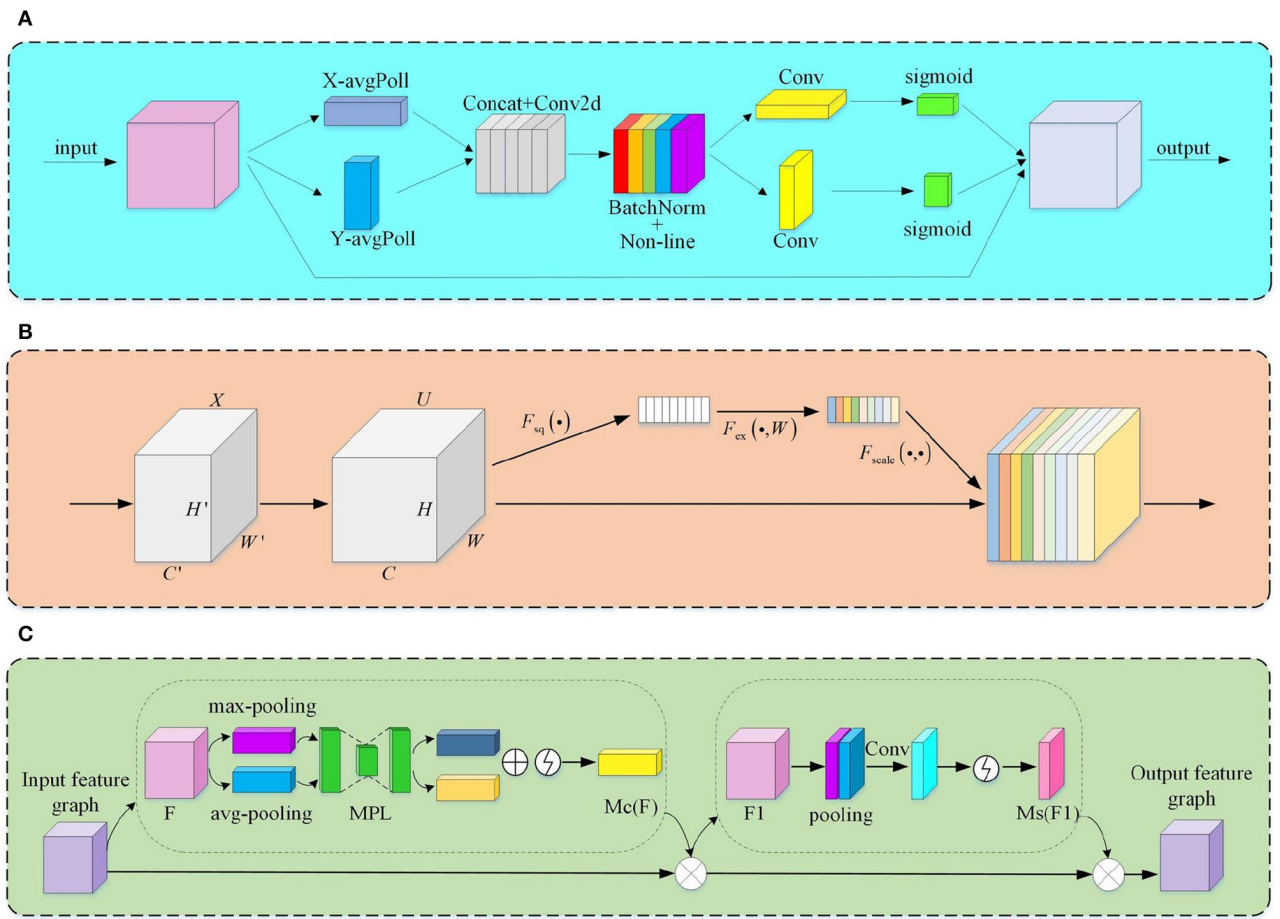
The module structure of the attention mechanism. **(A)** CA module; **(B)** SE module; **(C)** CA module.

Considering the limited context encoding capability of the Swin Transformer, we add the CA attention mechanism after the Patch Merging layer. CA attention decomposes the channel attention work process into two one-dimensional feature encoding processes and then performs feature aggregation along two directions in space. Figure 5A illustrates the structure of the CA attention mechanism. It first pools the feature maps globally averaged in two dimensions, height, and width, using convolution kernels of dimensions (H, 1) and (1, W), respectively:


(5) $$\begin{array}{l} \{\begin{array}{c} {\text{z}}_{c}^{h}\left(h\right)=\frac{1}{W}\underset{0\leq i<W}{\sum }x_{c}\left(h,i\right)\\ {\text{z}}_{c}^{h}\left(w\right)=\frac{1}{H}\underset{0\leq j<H}{\sum }x_{c}\left(j,w\right) \end{array} \end{array}$$

The above transformations obtain the feature maps in the space in the width and height directions, respectively. Then CA attention performs the stitching operation on the feature maps and performs the *F*_1_ transformation to obtain the feature map *f*. The formula is shown below:


(6) $$\begin{array}{l} f=\delta \left(F_{1}\left(\left[z^{h},z^{w}\right]\right)\right) \end{array}$$

Where *F*_1_ is the 1 × 1 convolutional transform function, [, ] denotes the splicing operation, and δ is the nonlinear activation function.

The feature map *f* is then convolved in the original height and width direction and activated by the Sigmoid activation function to obtain the feature map attention weights *g*^*h*^ and *g*^*w*^, which are given by the following equations:


(7) $$\begin{array}{l} \{\begin{array}{c} g^{h}=\sigma \left(F_{h}\left(f^{h}\right)\right)\\ g^{w}=\sigma \left(F_{w}\left(f^{w}\right)\right) \end{array} \end{array}$$

Where σ is the sigmoid activation function.

Finally, the CA attention mechanism is calculated by multiplicative weighting to obtain the output of the feature map with attention weights:


(8) $$\begin{array}{l} y_{c}\left(i,j\right)=x_{c}\left(i,j\right)\times g_{c}^{h}\left(i\right)\times g_{c}^{w}\left(j\right) \end{array}$$

It encodes long-range dependencies by precise positional information, enabling our model to utilize global contextual details efficiently. At the same time, CA has both channel and spatial domain attention mechanisms. Its introduction can better capture direction-aware and location-sensitive information for more accurate localization to identify objects of interest and improve feature representation.

### 3.3. Neck

#### 3.3.1. Improved CSPLayer—SECSP

CSPLayer (Wang et al., 2020) is mainly divided into two parts (Figure 1), a backbone part, which consists of shallow convolutional branches and sub-residual branches, and a residual part, which is directly connected to the output part of the CSPLayer through a simply processed 1 × 1 convolutional layer. The sub-residual bottleneck structure is an essential component of the CSPNet. It has a 1 × 1 convolutional stacked layer and a 3 × 3 convolutional stacked layer. Additionally, shortcut connections are applied to directly add elements to the output of the convolutional layer. The feature extraction process of the CSP network is primarily carried out in the sub-residual bottleneck structure, and its application significantly alleviates the gradient disappearance problem.

The use of CSPLayer makes the model over-consider the surrounding contextual information (Liu et al., 2022), which causes local information interference. To solve this problem, we introduce the SE attention mechanism in the Bottleneck module to selectively emphasize the feature information, weaken the interference information, and further enhance the focus on the target features. Meanwhile, the 3 × 3 convolutional layer in Bottleneck needs to deal with a large number of parameter operations while causing a large number of parameter redundancies. SE performs feature compression on the feature map down the spatial dimension, squeezing the global spatial information into the channel description. The output feature map z_*c*_ of channel **c** after compression is:


(9) $$\begin{array}{l} z_{c}=\frac{1}{H\times W}\overset{H}{\underset{i=1}{\sum }}\overset{W}{\underset{j=1}{\sum }}x_{c}\left(i,j\right) \end{array}$$

Where *x*_*c*_ is the input, *H* and *W* represent the two directions of height and width in space, respectively. This process dramatically reduces the redundant parameters in the network. Figure 5B illustrates the structure of SE.

#### 3.3.2. Improved up-sampling and down-sampling processes

The resolution of the feature maps at various sizes varies. Before feature fusion, down-sampling or up-sampling operations must shrink or enlarge the feature maps for feature fusion between feature maps of different scales. The process of compression and extension of feature maps brings about the problem of semantic information loss and the introduction of local interference. CBAM (as shown in Figure 5C) can focus on more profound feature semantic information by performing a hybrid pooling of both global average and global maximum over space and channels. Its introduction makes the model more robust. The specific working process is as follows:


(10) $$\{\begin{array}{ll} {\text{M}}_{\text{C}}\text{(F)}=\sigma \text{(}MLP(AvgPool\text{(F)})+MLP(MaxPool\text{(F)})\text{)} & \\ =\sigma {\text{(W}}_{\text{1}}{\text{(W}}_{\text{0}}{\text{(F}}_{\text{avg}}^{\text{c}}\text{)})+{\text{W}}_{\text{1}}{\text{(W}}_{\text{0}}{\text{(F}}_{\text{max}}^{\text{c}}\text{)))} & \\ {\text{M}}_{\text{S}}\text{(F)}=\sigma \text{(}f^{7\times 7}([AvgPool\text{(F); }MaxPool\text{(F) ​​}]\text{​​ })\text{)} & \\ =\sigma \text{(}f^{7\times 7}\text{( ​​}[\text{​​}{\text{ F}}_{\text{avg}}^{\text{s}}{\text{;F}}_{\text{max}}^{\text{s}}\text{ ​​}]\text{​​ ))} & \end{array}$$

Where M_C_(F) and M_*S*_(F) are one-dimensional and two-dimensional channel attention, respectively; σ is a sigmoid function; $W_{0}\in R^{C/r\times C}$, $W_{1}\in R^{C\times C/r}$; and *f*^7 × 7^ is a convolution kernel of 7 × 7 size.

**Figure 6:**
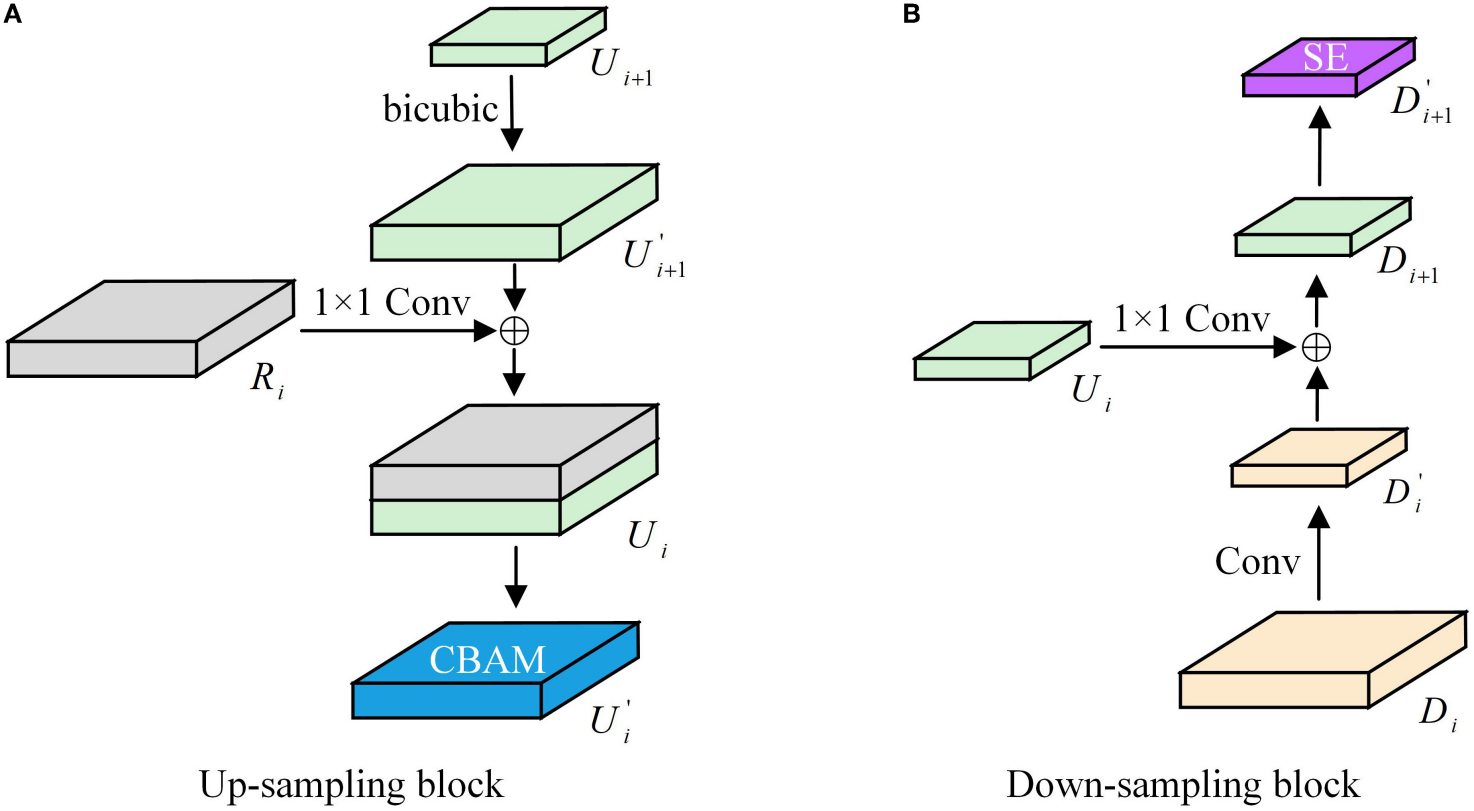
**(A, B)** Up/down-sampling structure diagram.

The deconvolution up-sampling approximation is considered the inverse operation of convolution. It can restore the feature map better by introducing training parameters for learning. However, this up-sampling method is prone to a tessellation effect, which causes pixel blocks to appear in the image. On the other hand, the interpolation method does not require any parameter learning. It performs predictive estimation of unknown points based on known pixel points, which can expand the size of the image and achieve the effect of up-sampling. Therefore, we use the bicubic interpolation algorithm for up-sampling instead of deconvolution, which reduces many parameter operations while preserving the original image information. Figure 6 illustrates the improved up-and-down sampling process.

#### 3.4. Head

Considering the fact that SAR images of ships in complex environments require a lot of feature information to identify targets, the commonly used coupled detection head will influence the model's performance and cannot detect the ship targets in SAR images of a complex environment. Therefore, the ST-YOLOX network model separates the classification and regression tasks by using a decoupled detection head for target detection to achieve the predicted output of target location and bounding box, which significantly increases the convergence speed and improves the accuracy of the model.

As for the loss function, SAR image ship detection is a single-class detection task. Hence, the loss function has only two components: localization loss (Reg) and confidence loss (Obj) (Jiang et al., 2022). The mathematical equations for these two components are as follows:


(11) $$\begin{array}{l} L\text{oss}=\frac{\lambda L_{reg}+L_{obj}}{N_{pos}} \end{array}$$

where λ is the balance coefficient; *N*_*pos*_ represents the Anchor Points quantity of positive samples; *L*_*obj*_ indicates the confidence loss; in our paper, the binary cross-entropy loss (BCE loss) is used as this loss function to promote numerical stability; *L*_*reg*_ represents the localization loss, and the Efficient-IOU (EIOU) (Zhang et al., 2022) loss function is used. Its mathematical expression is:


$$\begin{array}{lll} L & = & L_{IOU}+L_{dis}+L_{asp}=1-IOU+\frac{\rho^{2}\left(b,b^{gt}\right)}{c^{2}}+\frac{\rho^{2}\left(w,w^{gt}\right)}{c_{\omega }^{2}} \end{array}$$

(12) $$\begin{array}{ll} + & \frac{\rho^{2}\left(h,h^{gt}\right)}{c_{h}^{2}} \end{array}$$

where *L*_*IOU*_ is the overlap loss, *L*_*dis*_ is the center distance loss, and *L*_*asp*_ is the wide height loss. EIOU loss integrates the overlapping area, the distance to the center point, and the aspect ratio of the bounding box regression. It splits the loss term of the aspect ratio into the difference between the widths and the heights of the predicted and the minimum outer bounding boxes, which effectively solves the sample imbalance problem in the bounding box regression task, accelerates the convergence, improves the regression accuracy, and further optimizes the model.

With the aforementioned analysis, the ST-YOLOA detection model proposed in this paper applies to the detection of ship objects in SAR images under complex environments. It has the advantages of strong feature extraction capability, high utilization of high-level and low-level feature information, full information fusion, and robust performance, which is more suitable for ship target detection under realistic conditions.

## 4. Experiment

### 4.1. Experimental data and environment

#### 4.1.1. Dataset

In the paper, the experimental data are based on the publicly accessible SAR-Ship-Dataset (Wang et al., 2018) from the Key Laboratory of Digital Earth, Institute of Space and Astronomical Information, Chinese Academy of Sciences. The primary data sources of this dataset are Sentinel-1 SAR data and domestic Gaofen-3 SAR data, which use three polarization techniques: single-polarization, double-polarization, and full-polarization. It used 108-view Sentinel-1 and 102-view Gaofen-3 high-resolution SAR images to build a SAR ship target deep learning sample library containing 43,819 images of 256 × 256 pixels with 59,535 ship targets in total. The dataset contains a wide variety of ship types and backgrounds, including sea-surface scenes with noise interference from the ocean and ships of different scales and nearshore scenes influenced by complex backgrounds, such as islands, land constructions, and port terminals.

We used 4,000 photographs from the SAR-Ship-Dataset as the dataset for our experiment. The training set and the test set were randomly divided according to the ratio of 8:2, and 20% of the training set was randomly selected as the validation set. To increase the data diversity and ensure the model had a better training effect, we used two data enhancement methods, Mosaic (Tian et al., 2019) and Mixup (Zhang et al., 2017), to perform data enhancement operations on the dataset.

To test the ship detection capability of this model in complex environments, we selected 450 SAR ship images in complex environments, such as near-coastal ship targets affected by surrounding non-ship targets, ship targets with blurred or obscured imaging, ship targets with coherent speckle noise and complex background information, and multi-scale ship targets. We named the two SAR ship detection test sets constructed as norm test set (NTS) and complex test set (CTS), respectively, and combined the two sets into one merged test set (MTS). We used the combined performance of the model in these three test sets as the criterion to verify its detection ability. The details of the ship data set used for the experiments are shown in Table 1.

**Table 1:** Experimental data set details.

**Dataset**		**Number of images**	**Target background**	**Number of ships**
Train		2,560	General background	3,440
Val		640	General background	843
Test	NTS	800	General background	1,020
	CTS	450	Complex background	943
	MTS	1,250	General Background + Complex background	1,963

#### 4.1.2. Evaluation indicators

This paper chooses the average precision (AP) (Everingham et al., 2009) as the main evaluation index to assess the effect of SAR image ship detection. It contains two parameter metrics, Precision and Recall. The calculation formula is:


(13) $$\begin{array}{l} \{\begin{array}{c} P=\frac{TP}{TP+FP}\\ R=\frac{TP}{TP+FN}\\ AP=\overset{1}{\underset{0}{\int }}P\left(r\right)dr \end{array} \end{array}$$

where *TP* (true positive) is the number of ships marked as ship targets, *FN* (false negative) is the number of ship targets marked as non-ships, *FP* (false positive) is the number of non-ships marked as ship targets, and *P(r)* is the area under the PR curve with precision and recall, which is AP.

Also, to better measure, the comprehensive performance of the model, Parameters, GFLOPs, and FPS are introduced as evaluation metrics in this paper.

#### 4.1.3. Experimental environment and parameter setting

In this paper, the experimental environment was based on Linux system architecture, using the Ubuntu 18.04 operating system, equipped with an Intel(R) Core i9 10980 XE CPU and NVIDIA RTX 2080TI graphics card with 11 GB video memory. The deep learning framework used PyTorch, with accelerated training via CUDA 10.1 and cuDNN 7.6.

In this paper, the experimental hyperparameters are referred to the literature (An et al., 2019; Yuan and Zhang, 2021; Wu et al., 2022), and the main settings are as follows: setting the training period to 300 epochs, the maximum learning rate of the model to 0.01, and the minimum learning rate to 0.0001. The optimizer was stochastic gradient descent (SGD), and the weights decayed at a rate of 0.0005. To increase the speed of data reading, we employed multi-threaded data reading and used cosine (COS) as the learning rate descent method. The test was run with the following parameters: a non-maximum suppression threshold of 0.65, a confidence level of 0.001, and a prediction probability threshold of 0.5.

### 4.2. Ablation experiments

To validate the performance of each major module and loss function in the ST-YOLOA model, we performed ablation experiments. In these experiments, as the benchmark model, we used the YOLOX network, which through eight groups of networks with various structures were used to test the effects of various strategies on the detection effectiveness of the model. These strategies included changing the Swin Transformer backbone network; adding CA, SE, and CBAM attention mechanisms; modules; and using the EIOU loss function. We used the same experimental equipment and training parameters in each experiment to test and validate the detection effect in the NTS, CTS, and MTS. Table 2 shows the results of the ablation experiments.

**Table 2:** The ablation experiments results.

**Serial number**	**Swin-T**	**Attention**	**EIOU loss**	**AP/%**			**FPS**	**GFLOPs**	**Parameters**
				**NTS**	**CTS**	**MTS**			
1	-	-	-	96.23	70.86	85.52	**22.74**	**27.27**	**8.94**
2	√	-	-	96.30	73.81	86.72	22.24	89.60	32.72
3	-	√	-	96.94	71.64	86.37	20.24	27.28	9.00
4	-	-	√	97.23	73.61	88.27	*22.38*	**27.27**	**8.94**
5	√	√	-	96.71	74.19	87.74	21.60	89.61	32.77
6	√	-	√	97.28	*74.40*	88.16	22.00	89.60	32.72
7	-	√	√	**97.81**	72.40	86.93	20.49	27.28	9.00
8	√	√	√	*97.37*	**75.69**	**88.50**	21.40	89.61	32.77

Bold indicates the best result of each column, italic is the second best result; “-” is no module added, “√” is the module added.

The benchmark model YOLOX network is the least efficient, as seen in Table 2. Comparing the ordinal number 2 in the table, we can see that the improvement in the average accuracy AP on the NTS using the Swin Transformer backbone is insignificant. However, the improvement in the detection of CTS is significant. Swin Transformer contains a large number of parameters but does not have much impact on the detection speed. Its introduction gives the model an AP improvement of 2.95% with almost no loss in detection speed FPS. This demonstrates that Swin Transformer can focus on global information, particularly for the extraction of sophisticated features with a significantly enhanced effect. Serial number 3 adds CA, SE, and CBAM attention mechanism modules to the feature extraction and feature fusion sections, improving the AP of the NTS and the CTS by 0.71 and 0.78%, respectively, while the FPS decreases by 2.5 frames per second, showing that this method is capable of adaptively focusing on and using useful local feature information to lower the rate of missed detection but complicates the model computationally and structurally. Serial number 4 introduces the EIOU loss function and achieves good detection results on both test sets, with an AP improvement of 1.00 and 2.75%, respectively. Although the FPS decreases slightly, this still demonstrates that the introduction of the EIOU loss enhances convergence speed and prevents the degradation of model training caused by the uneven distribution of positive and negative samples, which is a successful addition strategy. The trials in serial numbers 5 to 8 are composite multi-strategy experiments. A comparison of serial numbers 2 to 4 shows that the use of a variety of tactics together achieves better results than the use of just one strategy. From serial numbers 5 and 6, it can be seen that Swin Transformer effectively addressess any speed reduction brought on by the addition of other modules. Serial number 7 uses both the attention module and EIOU loss to ensure that the model works optimally on the NTS. Serial number 8 is the ST-YOLOA network model proposed in this paper. Compared with YOLOX, its AP is improved by 1.14 and 4.83% in the NTS and the CTS, respectively. Although the effect is slightly reduced compared to serial number 7 on NTS, it still achieved second place in the comparison experiment. Its detection speed and detection effect in a complex environment are also substantially ahead.

**Figure 7:**
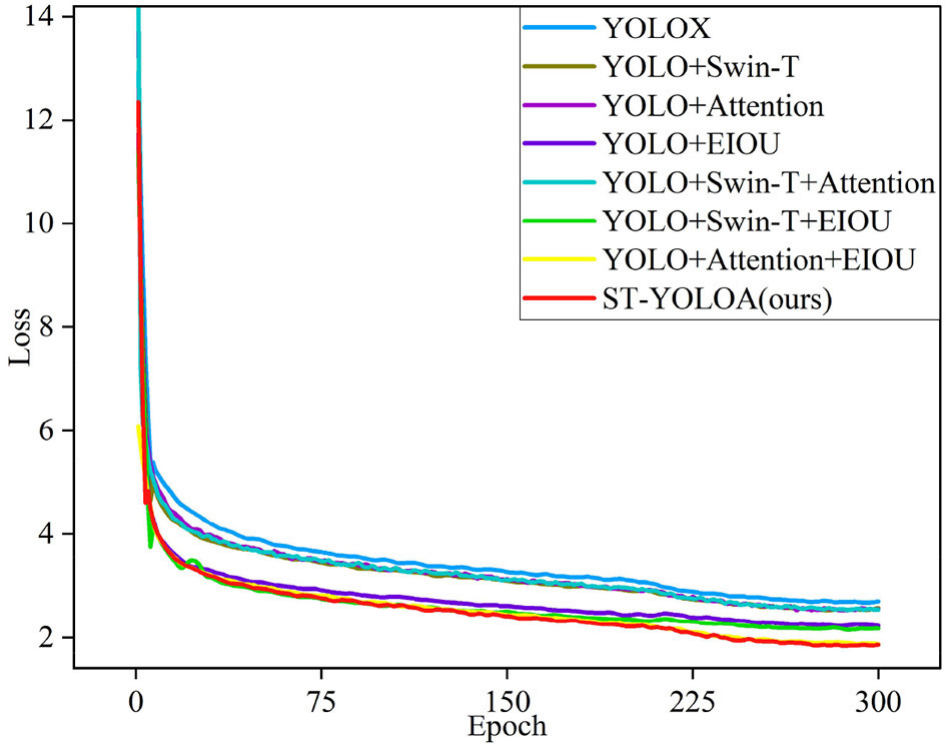
Loss function curves of the ablation experiments.

The loss function curve in Figure 7 clearly illustrates our algorithm's superior performance. In conclusion, the model in this paper meets the real-time detection criteria, while displaying substantially improved target detection accuracy, especially showing excellent detection performance in the complicated environment of the dataset.

In this paper, we introduce three attention mechanisms to enhance the network performance according to the characteristics of different modules to enhance the focus on ship targets. We conducted ablation experiments on three attentional mechanisms, SE, CA, and CBAM, to validate the effectiveness of each attentional mechanism. Table 3 shows the experimental results. The results show that the combination of the three attention mechanisms works optimally.

**Table 3:** Ablation experiments of attentional mechanisms.

**SE**	**CA**	**CBAM**	**Precision/%**	**Recall/%**	**AP/%**	**FPS**	**GFLOPs**	**Parameters**
			79.87	83.49	86.72	22.24	89.60	32.72
√			84.83	81.46	87.46	21.94	89.60	32.72
√	√		85.71	82.53	87.64	21.87	89.60	32.75
√	√	√	**86.27**	**83.61**	**87.74**	21.60	89.61	32.77

Bold represents the maximum value in each column. √ represents the module that added one row of that column to the network.

### 4.3. Comparative experiments

To objectively evaluate the detection effectiveness of the ST-YOLOA model, we performed comparative experiments using our model and other existing target detection methods. The range of comparison algorithms covers a wide range, among which CenterNet, Faster R-CNN, SSD, RetinaNet (Lin et al., 2017b) and EfficientDet (Tan et al., 2020) are classical target detection models, YOLOv5 (Jocher, 2020) and YOLOX are newly published high-performance target detection models in recent years, and YOLOv7 (Wang et al., 2022) is one of the most advanced detection models at present. Table 4 displays the results of the comparison experiments.

**Table 4:** Performance comparison of the different algorithms.

**Algorithm**	**NTS**			**CTS**			**MTS**			**FPS**	**GFLOPs**	**Parames**
	**P/%**	**R%**	**AP/%**	**P/%**	**R/%**	**AP/%**	**P/%**	**R/%**	**AP/%**			
CenterNet	*94.97*	85.20	96.22	**82.64**	57.05	70.95	**89.85**	71.68	85.54	25.13	109.34	32.67M
Faster R-CNN	61.84	**98.33**	95.98	38.88	**76.35**	65.03	50.04	**87.72**	83.18	25.47	401.91	136.69M
SSD	90.40	85.88	94.43	68.68	57.90	66.48	80.61	72.44	82.83	**50.40**	273.40	23.61M
RetinaNet	92.82	91.27	96.80	72.23	64.26	71.36	83.44	78.30	86.47	39.15	163.49	36.33M
EfficientDet	**95.60**	87.35	96.81	*81.33*	55.89	72.32	*89.75*	72.24	86.95	*45.64*	**7.40**	**3.83M**
YOLOv5	90.86	90.69	95.65	69.28	63.63	67.03	80.94	77.69	83.54	32.88	*16.38*	*7.06M*
YOLOX	88.79	94.71	96.23	72.77	67.44	70.86	82.66	82.32	85.52	22.74	27.27	8.94M
YOLOv7	92.06	95.49	*97.36*	75.70	68.40	*73.56*	84.76	82.48	87.50	34.60	105.11	37.20M
ST-YOLOA	91.82	*95.78*	**97.37**	74.24	*72.75*	**75.69**	84.04	*83.44*	**88.50**	21.40	89.61	32.77M

Bold font denotes the best outcome for each column, italics is the second best result.

CenterNet predicts the bounding box by learning the centroid and corner point pairs in the feature map without relying on the predetermined anchor box. It has the highest precision rate but a poor recall rate. As a representative algorithm of two-stage detection, the Faster R-CNN detection recall rate has improved significantly but the detection accuracy can be still improved. SSD has been experimentally shown to be unable to efficiently detect ships in complicated surroundings in a SAR picture, despite having a higher detection speed and a more condensed network model. RetinaNet surpasses the previous two-stage algorithm in terms of accuracy and the last detection single-stage algorithm in terms of speed, but there is still much room for improvement efficientDet, as a lightweight network model, has the lowest number of parameters and computations, but its accuracy still needs further improvement. YOLOv5 and YOLOX, as new single-stage detection models, have fewer parameters and computation and more concise network models, which perform better than traditional detection algorithms. However, there is still a certain degree of false detection and leakage, with serious false detection and leakage problems in complex conditions. YOLOv7 shows excellent performance in speed and accuracy, but the detection capability for complex backgrounds still needs further improvement. Compared with other models, ST-YOLOA has an average number of parameters and computation and has higher detection accuracy. It can also meet the basic requirements of real-time detection. Therefore, ST-YOLOA has good overall performance in terms of comprehensive detection, particularly when it comes to SAR image ship detection in complicated environments.

**Figure 8:**
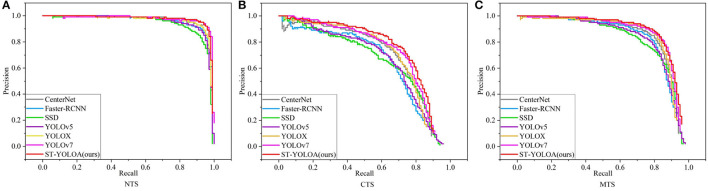
Comparison of the PR curves of different algorithm models. **(A)** NTS; **(B)** CTS; **(C)** MTS.

In this research, to demonstrate the detection performance of various models, we compared the PR (precision–recall) curves of each model on two separate test sets. The PR comparison curves are displayed in Figure 8.

**Figure 9:**
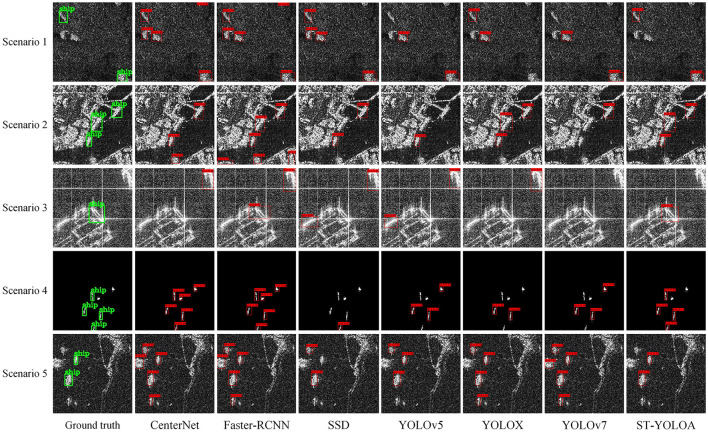
Comparison of the detection results.

To visually compare and analyze the detection effects of the ST-YOLOA model and other algorithms in different scenarios, we selected SAR images containing near-coast and far-sea ship targets. Figure 8 shows the detection effect, where the first column of Figure 9 shows the actual labeling result, and other columns show the detection results of each algorithm.

### 4.4. Generalization ability test

In this paper, to illustrate the generalization capacity of ST-YOLOA, we used two distinct ways for partitioning data (Guo W. et al., 2022): (1) Partitioning the data at random into five ratios: {9:1, 8:2, 7:3, 6:4, 5:5}. (2) Partitioning the data multiple times at random into the ratio 8:2. The test results of the two methods of dataset division are provided in Tables 5, 6.

**Table 5:** Sample cutting in different proportions.

**Proportioning**	**Precision %**	**Recall %**	**AP %**
9:1	92.17	95.84	97.24
8:2	91.82	95.78	97.37
7:3	91.92	96.92	97.44
6:4	91.38	96.28	97.27
5:5	91.70	96.23	96.98
Mean	91.798	96.21	97.26
Variance	0.08452	0.2078	0.03085

**Table 6:** Multiple sample cuts in the same proportion.

**Cutting times**	**Precision %**	**Recall %**	**AP %**
1st	91.63	96.60	97.39
2nd	91.82	95.78	97.37
3rd	91.69	96.39	97.27
4th	92.00	96.51	97.24
5th	91.47	97.05	97.36
Mean	91.722	96.266	97.326
Variance	0.03997	0.11733	0.00443

Table 5 shows that although the number of samples of ship targets in the test samples that are randomly divided by different ratios of the dataset varies significantly, the average accuracy of ST-YOLOA does not change much. However, even though the average accuracy of the samples divided in the ratio of 5:5 among them differed more than the others, it exhibits a good detection ability, which is analyzed because the detection effect degrades as a result of an insufficient number of training samples. The variance of AP for each sample in this experiment is 0.03085, and the variances of the precision and recall are 0.08452 and 0.2078, respectively. This indicates that the ST-YOLOA model proposed in this paper has a stable detection effect for test sets with different numbers of data samples and shows a strong generalization ability.

Due to the same number of samples and smaller variations in the number of ship targets, as shown in Table 6, the variance of the experiment's indicators is lower for samples divided multiple times at the same scale. The mean and variance of the SA-YOLOA model for the same proportion of samples divided multiple times were 91.722% and 0.03997 for precision, 96.266% and 0.11733 for recall, and 97.326% and 0.00443 for mean precision, respectively.

The information in Tables 5, 6 leads to the conclusion that ST-YOLOA performs well and has excellent generalization capacity, both in test samples with various ratios of randomly divided datasets and in test samples with the same proportion of multiple divided datasets.

### 4.5. Detection effect of the ST-YOLOA model in different scenarios

**Figure 10:**
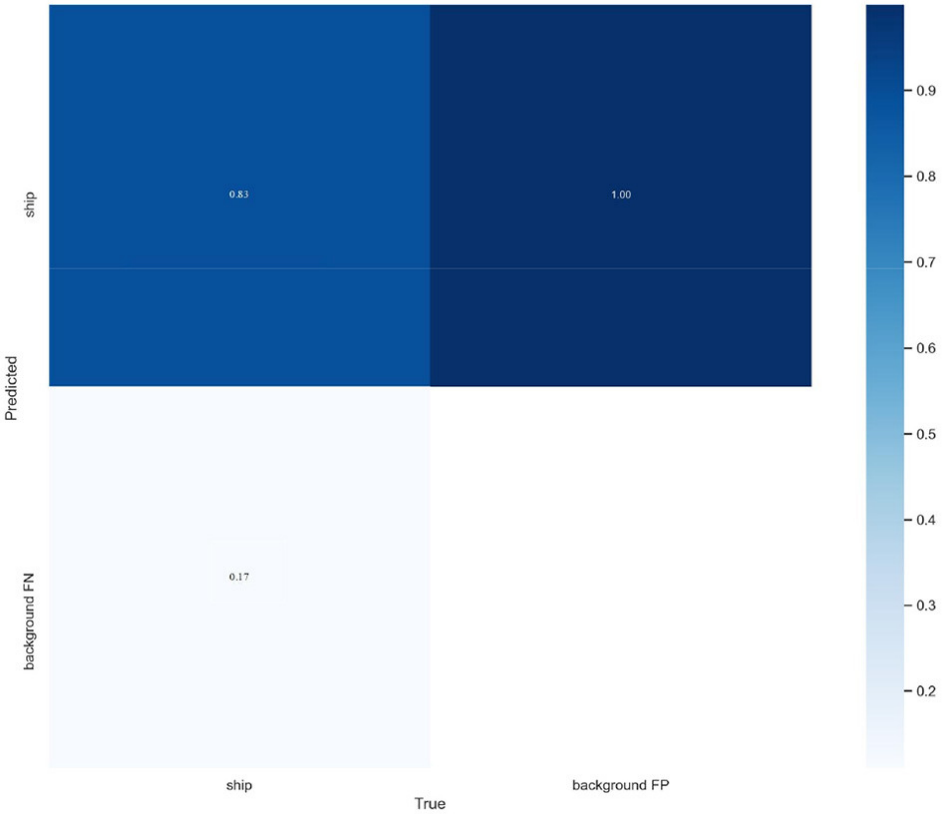
Confusion matrix.

To visualize the detection effect of the ST-YOLOA model and further measure the model performance, this section first shows the schematic of the confusion matrix of the ST-YOLOA algorithm. As shown in Figure 10, the confusion matrix demonstrates that ST-YOLOA has good performance.

**Figure 11:**
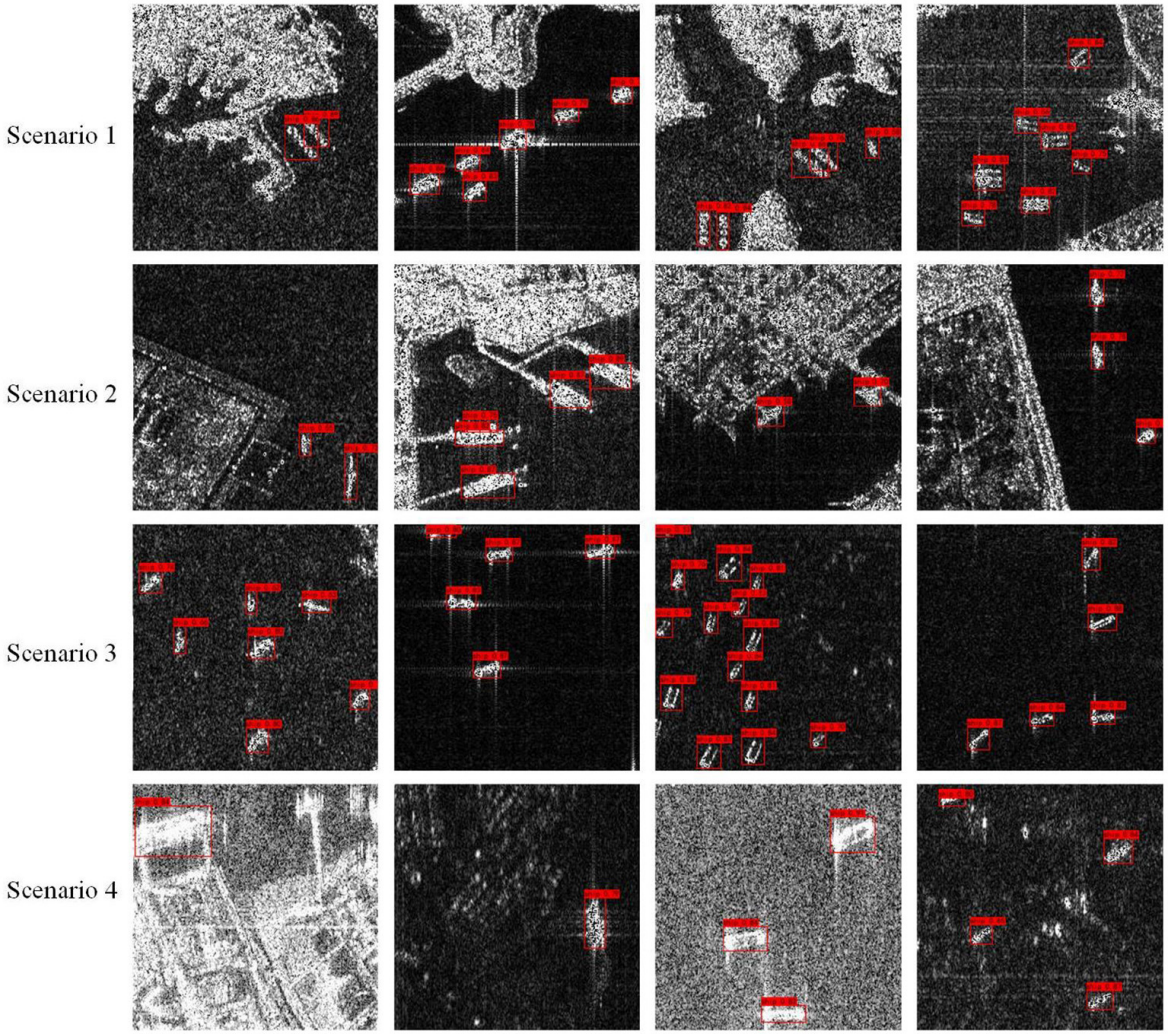
Detection effects in different scenarios.

In this study, we demonstrate the effect of ship target detection under different scenarios and scales, including near-shore and far sea. Figure 11 presents the detection effect in each scenario. The first and second rows are near-shore ship targets near islands and near-shore buildings, respectively. Such targets have complex backgrounds and are susceptible to the influence of other non-ship targets around them. Multiple near-shore ship targets can easily be framed by a single detection box due to the dense docking of ships, which suppresses candidate boxes with high overlap and low prediction scores. The third row is a small, dense target in the distance that is easy to miss because it has a small ship scale. The ship target in the fourth row is prone to erroneous target localization since it has indistinct target borders and complicated background information. In all four aforementioned scenarios, the ST-YOLOA model significantly improved the detection rate and accuracy, as can be seen from the figure, and produced positive detection results.

### 4.6. Limitations and discussion

The results of previous experimental studies show that our model achieves sound visual effects in SAR ship detection in complex scenes. It is demonstrated that the ST-YOLOA model can learn global features and can be used to extract more powerful semantic features for ship target detection in harsh environments and complex scenes. However, our approach still suffers from some limitations.

The relatively high computational complexity and large number of parameters of the Swin Transformer module lead to more extended training and inference time. As seen from the experimental ablation results in Table 1, although we have used the Swin-Transformer network with a smaller model as much as possible, its use still introduces many parameters compared to the base model. The Swin Transformer network has a solid global modeling capability, capturing rich global feature information and integrating global data. This process requires a vast amount of support operations, resulting in more parameters and computations than other models. At the same time, the computational complexity of the Swin Transformer increases with the length of the input sequence. When dealing with very long input sequences, Swin Transformer may face problems such as high computational complexity and large memory consumption, which need to be alleviated by using lightweight models or other techniques.

## 5. Conclusions

To ensure the accuracy of SAR ship target recognition under complicated situations, in this study, we have suggested a more extended ST-YOLOA ship target identification model. To begin with, the feature extraction section adds the Patch Embedding module after the input layer to chunk and flatten the input image and then produces feature maps of varying sizes using Swin Transformer Blocks and the Patch Merging layer. A coordinated attention mechanism is designed at the end to simultaneously capture position information and channel relationships, which significantly improves the performance of downstream tasks. Second, to effectively use semantic and localization information, the PANet is employed to thoroughly fuse high-level and low-level feature information. Finally, a decoupled detection head in the target detection section is used to significantly speed up model convergence and improve the position loss function, both of which improve model performance. This model is more suited for ship target detection in challenging surroundings and complex circumstances because it can extract more potent semantic characteristics and can better learn global features than other detection models.

Considering that our model focuses on improving SAR ship detection accuracy in complex environments, the vital index of the number of parameters of the model is ignored to a certain extent. In the future, we will further conduct model optimization and carry out research on model lightweight by adjusting hyperparameters and model compression methods, such as quantization, distillation, and pruning, and further analysis on lightweight Swin Transformer to achieve lower model parameter computation, faster training speed, and maintain previous accuracy.

## Data availability statement

The original contributions presented in the study are included in the article/supplementary material, further inquiries can be directed to the corresponding author.

## Author contributions

Conceptualization: XY, KZ, and RL. Methodology and writing–original draft preparation: KZ and RL. Software: RL. Investigation: JF and SW. Resources and visualization: KZ. Writing–review and editing: XY, KZ, and SW. Supervision: KZ and SW. Project administration and funding acquisition: XY. All authors have read and agreed to the published version of the manuscript.
